# Enhancement of the Bioavailability and Anti-Inflammatory Activity of Glycyrrhetinic Acid via Novel Soluplus^®^—A Glycyrrhetinic Acid Solid Dispersion

**DOI:** 10.3390/pharmaceutics14091797

**Published:** 2022-08-26

**Authors:** Hao Wang, Runwei Li, Yuan Rao, Saixing Liu, Chunhui Hu, Yong Zhang, Linchao Meng, Qilin Wu, Qiuhong Ouyang, Hao Liang, Meng Qin

**Affiliations:** 1 Beijing Advanced Innovation Centre for Soft Matter Science and Engineering, State Key Laboratory of Chemical Resource Engineering, College of Life Science and Technology, Beijing University of Chemical Technology, Beijing 100029, China; 2 Medical College, Qinghai University, Xining 810001, China; 3 Technology Corporation, Beijing 100029, China; 4 Star Health Botanical Technology Corporation, Beijing 100029, China

**Keywords:** glycyrrhetinic acid, Soluplus^®^, solid dispersions, anti-inflammatory, biosafety, bioavailability

## Abstract

Glycyrrhetinic acid (GA) is an anti-inflammatory drug with potential for development. However, the poor solubility of GA in water leads to extremely low bioavailability, which limits its clinical applications. Solid dispersions have become some of the most effective strategies for improving the solubility of poorly soluble drugs. Soluplus^®^, a non-cytotoxic amphiphilic solubilizer, significantly improves the solubility of BCS II drugs and improves the bioavailability of insoluble drugs. l-arginine (L-Arg) can be used as a small molecular weight excipient to assist in improving the solubility of insoluble drugs. In this study, we developed a new formulation for oral administration by reacting GA and L-Arg to form salts by co-solvent evaporation and then adding the polymer-solvent Soluplus^®^ with an amphiphilic chemical structure to prepare a solid dispersion GA-SD. The chemical and physical properties of GA-SD were characterized by DLS, TEM, XRD, FT-IR and TG. The anti-inflammatory activity of GA-SD was verified by LPS stimulation of RAW 267.5 cells simulating a cellular inflammation model, TPA-induced ear edema model in mice, and ethanol-induced gastric ulcer model. The results showed that the amide bond and salt formation of GA-SD greatly improved GA solubility. GA-SD effectively improved the anti-inflammatory effect of free GA in vivo and in vitro, and GA-SD had no significant effect on liver and kidney function, no significant tissue toxicity, and good biosafety. In conclusion, GA-SD with L-Arg and Soluplus^®^ is an effective method to improve the solubility and bioavailability of GA. As a safe and effective solid dispersion, it is a promising anti-inflammatory oral formulation and provides some references for other oral drug candidates with low bioavailability.

## 1. Introduction

Inflammation is the first protective response in biological systems and can protect against injury caused by harmful stimuli, such as physical insults, chemical insults, or infection [1,2]. However, an excessive inflammatory response can damage normal tissues [3]. Due to low or no toxicity, characteristics of natural biologically active substances can greatly reduce the risk of complications in treating excessive inflammation [4]. Therefore, the public is increasingly interested in the use of traditional plant-based medicines to prevent and treat inflammatory diseases. This trend has prompted the further development of natural products with anti-inflammatory properties. A variety of plant extracts have been scientifically shown to exhibit anti-inflammatory activity by modulating the expression levels of inflammation-related genes [5,6].

Glycyrrhetinic acid (GA) is a triterpene saponin generated by the hydrolysis of glycyrrhizic acid to remove the sugar and acid chains and is the main active ingredient in licorice. According to the Biopharmaceutical Classification System, GA is a type II drug [7]. GA has shown a variety of pharmacological actions, such as antiallergic, antimicrobial, antiviral, antihepatotoxic and antitumor activities [8]. Many studies have further confirmed the good immunomodulatory effects of GA. Peng et al. [9] found that GA could modulate the innate immune response by binding to the TLR4 receptor to induce the expression of its downstream signaling molecules. Wang et al., demonstrated that GA can exert anti-inflammatory effects by inhibiting the activity of PI3Kp110δ and p110γ in the PI3K/Akt/GSK3β signaling pathway, blocking Ser phosphorylation of the p65 subunit, a key subunit required for NF-kB activation, indirectly inhibiting NF-kB activation and reducing the release of downstream inflammatory cytokines. GA also promotes dissociation of a glucocorticoid receptor-heat shock protein 90 (GR-HSP90) complex and inhibits excessive inflammatory responses [2,5]. GA is an anti-inflammatory drug with developmental potential. However, it is composed of two hydrophilic glucuronic acid residues and one hydrophobic glycogen, granting it high hydrophobicity and low solubility in aqueous solutions and pH-dependent solubility [10]. Its lower solubility makes it less bioavailable, limiting its clinical applications [11]. Therefore, the use of appropriate strategies to increase the solubility of GA will greatly improve the bioavailability of GA and enhance the clinical efficacy of GA for the treatment of inflammatory diseases.

Solid dispersions have become some of the most effective strategies for improving the solubility of poorly soluble drugs [12,13]. The drug can be dispersed in the polymer, which may have a higher porosity, reduced particle size and improved wettability [13]. The choice of polymer in a solid dispersion can help prevent the recrystallization of the drug [14]. Sui and Chu et al., investigated the effect of polyvinylpyrrolidone (PVP) as a GA solid dispersions carrier at different molecular weights, investigating the dissolution behavior and physicochemical properties. PVP-GA-SDs displayed a good enhancement of dissolution rate and equilibrium solubility compared with pure drugs and corresponding physical mixtures [15]. Dong et al., prepared ternary solid dispersion (TSD) systems containing alkalizers to increase the dissolution of GA; GA-TSD significantly improved the solubility and dissolution of GA [16]. These studies confirmed that the preparation of solid dispersion is one of the important ways to improve the solubility of GA. However, there are few studies on the preparation of GA solid dispersion, and discussions on drug loading, entrapment efficiency, and anti-inflammatory effect of solid dispersion are incomplete. Polyethylene caprolactam-polyvinyl acetate-polyethylene glycol graft copolymer (Soluplus^®^) is a noncytotoxic amphiphilic solubilizer, absorbent, and penetrant that can be used as a carrier in the formulation of solid dispersions [17]. The bifunctional character of Soluplus^®^ allows it to act as a matrix polymer for solid solutions and to increase the solubility of insoluble drugs [18]. The low critical micelle concentration of Soluplus^®^ is 6.63 × 10^−3^ mg/mL [19]. When the concentration of Soluplus^®^ exceeds the low critical micelle concentration, Soluplus^®^ encapsulates the hydrophobic drug in a hydrophobic core where the carbonyl group in the molecular structure binds to the hydroxyl group of the drug. At the same time, Soluplus^®^ prevents crystal formation by reducing the energy of the particle surface, further improving the stability of the micelles [20,21]. Studies have shown that Soluplus^®^ can significantly increase the dissolution of BCS II drugs and improve the bioavailability of insoluble drugs [22]. L-arginine (L-Arg) can be used as a small molecular weight excipient and undergo specific molecular interactions with drugs to produce an amorphous material, thus, assisting in improving the solubility of insoluble drugs [23]. These interactions include salt formation between L-arginine and the drug, which changes the pH, improves the solubility of pH-dependent drugs, and controls the aggregation of drug crystals [24]. It has been demonstrated that L-Arg can act as an alkalizer to form salts with glycyrrhetinic acid through strong electrostatic attraction, which can significantly improve the solubility of pH-dependent drugs [16].

This study aimed to prepare solid dispersions with L-Arg as an excipient and Soluplus^®^ as a polymer to improve the solubility and bioavailability of free GA and provide a new scheme for the preparation of GA-SD. The effects of GA-SD on the anti-inflammatory activity of free GA were analyzed in an LPS-stimulated cellular inflammation model, a mouse ear edema model and an alcohol-induced gastric ulcer model. The biosafety of GA-SD was also demonstrated. The results showed that the GA-SD solid dispersion system developed in this study is promising for improving the bioavailability and anti-inflammatory effects of free GA. With the improvement of the solubility and utilization of GA, the application scope of GA in the treatment of many inflammatory diseases will be expanded.

## 2. Materials and Methods

### 2.1. Materials

GA was purchased from Beijing Mairuida Technology Co., Ltd., Soluplus^®^ was purchased from BASF (Ludwigshafen, Germany), and L-Arg was purchased from Beijing Aibifan Biotechnology Co. 12-O-tetradecanoylphorbol-13-acetate (TPA) (p1585, Sigma-Aldrich, Munich, Germany). All other related chemicals and reagents were analytically pure.

### 2.2. Animals and Cell Line

The mouse macrophage RAW264.7 cell line was purchased from the Cell Resource Center of Peking Union Medical College Hospital. RAW264.7 cells were cultured in DMEM complete medium (Gibco™, Grand Island, NY, USA) containing 10% fetal bovine serum (Gibco^TM^, Sydney, Australia) at 37 °C in a 5% CO_2_ incubator and passaged by gently scraping the adherent cells with a cell scraper at a ratio of 1:6–1:8.

Experimental animals: KM mice (18~22 g, male) were purchased from Beijing Huafukang Biotechnology Co. All animals were kept under stable temperature conditions (20–26 °C) and 12 h light/dark cycles for three days. All experiments were conducted by the regulations of the National Institutes of Health (NIH) and the Beijing Laboratory Animal Ethics Committee.

### 2.3. Fabrication of GA-SD

To prepare GA-SD, the co-solvent evaporation method was used [25,26]. Briefly, solutions of GA, L-Arg and Soluplus^®^ were prepared in the proportions shown in Table S1. GA was dissolved in 50 mL of anhydrous ethanol, L-Arg was dissolved in 50 mL of deionized water, and both were ultrasonicated to dissolve them completely. The GA and L-Arg solutions were stirred on a magnetic stirrer at room temperature until thoroughly mixed and then left to stand for 30 min to allow GA to salt with L-Arg. The mixture was spin-dried at 40 °C and 100 rpm. The Soluplus^®^ solution was prepared with 50 mL of deionized water, and the sample was redissolved by adding the prepared Soluplus^®^ solution to the rotary dried sample. The hydrophilic L-Arg fraction allowed the GA-L-Arg complex to disperse in water and the hydrophobic GA fraction combined with the hydrophobic structure of Solulplus^®^ to form the GA-L-Arg-Solulplus^®^ ternary complex. The resulting solution was a GA solid dispersion. The samples were freeze-dried to obtain a solid dispersion powder.

### 2.4. Drug Loading Analysis

In this study, the drug loading capacity of the prepared GA-SD was tested to screen for the optimal reagent ratio for the preparation of solid dispersions. GA solid dispersions prepared under different mass ratios of substances were first taken and dissolved in deionized water, and then the drug loading was detected by HPLC under the following conditions: phase A methanol, phase B 0.2% phosphoric acid, A:B = 90:10, flow rate 1 mL/min; wavelength 250 nm, column temperature 35 °C; each injection volume was 20 µL.

### 2.5. Characterizations of GA-SD

#### 2.5.1. Particle Size and Zeta Potentials

A Nano-ZS 2000 (Malvern Instruments Ltd., Malvern, UK) was used to determine the particle size and Zeta potential. Before measurement, an appropriate amount of Soluplus^®^ and GA-SD were weighed respectively and configured into a 2 mg/mL solution. All the samples were diluted 3 times with deionized water at a ratio of 1:3 (*v*/*v*), and then the average particle size and Zeta Potentials were measured by a nanoparticle size potential analyzer.

#### 2.5.2. Transmission Electron Microscopy

Morphological observation of GA-SD was performed using a Hitachi-HT7700 (120 kV) transmission electron microscope. Double distilled water was used to dilute the GA-SD powder and ultrasonically disperse it for 30 min. A drop of diluent (approximately 5 μL) was loaded onto a copper mesh (200 mesh, EMS) coated with formaldehyde/carbon, and the sample was air-dried before observation.

#### 2.5.3. Fourier Transform Infrared Spectroscopy

Infrared analysis was performed to identify the formation of complexes by evaluating deviations in peak shape, position, and intensity. A certain amount of GA, Soluplus^®^, L-Arg, a physical mixture of the three, and GA-SD powder were scanned in the range of 4000–400 cm^−1^ by FT-IR (Thermo Scientific Nicolet iS20).

#### 2.5.4. X-ray Diffraction

To observe the state of the GA before and after wrapping, the GA-SD powder was characterized by X-ray crystal diffraction (XRD) (Pert3 Powder). Certain amounts of GA, Soluplus^®^, L-Arg, their physical mixture and GA-SD powder were taken and laid flat on a sample table and then analyzed by XRD. The scanning speed was 5°/min, and the scanning range was 5–80°.

#### 2.5.5. Thermal Gravimetric Analysis

Thermogravimetric analysis (TA SDT 650) was employed to evaluate the thermal stability of GA-SD. The samples of about 10 mg were sealed in an aluminum plate and heated from 30 °C to 800 °C with the rate of 10 °C/min under an atmosphere of nitrogen. The weight variation of the samples was recorded in relation to temperature.

#### 2.5.6. Stability Studies

To investigate the stability of the prepared GA-SD, we took 50 mg of the prepared GA-SD powder and dissolved it in 25 mL of deionized water. The studies were carried out at room temperature for 63 days. The change in GA content in the solid dispersion was measured by HPLC every 7 days. Meanwhile, the GA-SD were stored at 4 °C for 7 months and analyzed by X-ray crystal diffraction to determine the crystallization propensity of GA-SD over time.

#### 2.5.7. Solubility of Formulation

The GA-SD (500 mg) was respectively added to a 15 mL centrifuge tube containing 2 mL deionized water and then shaken at 37 °C for 72 h. After the shaking, the samples were filtered with a 0.22 filter membrane and diluted with absolute ethanol. Then, the GA was quantified by HPLC (the conditions were described in Section 2.4). Each experiment was conducted in triplicates and the average solubility was obtained.

### 2.6. In Vitro Anti-Inflammatory Activity

The safe concentration of cell administration on RAW264.7 cells was assessed by CCK-8 assay. Briefly, cells dispersed evenly in the medium were seeded at a density of 5.0 × 10^3^ cells/well in 96-well plates. The next day, cells were treated with different doses (0, 12.5, 25, 50, 100, 200, and 300 µM) of GA and GA-SD, respectively, for 24 h. Then, CCK-8 solution was added and the optical density (OD) at 450 nm was determined. GA was dissolved in DMSO, and the volume of DMSO used was one-thousandth of the volume of the medium [27]. The dissolved GA was configured with DMEM serum-free medium to a concentration of 50 µM. The total mass of GA-SD required for drug-loaded 50 μM GA was calculated according to the drug loading of solid dispersion of 24.8%. Then the serum-free DMEM was prepared, filtered through a 0.22 µm filter to sterilize the preparation, and then stored at 4 °C.

RAW264.7 cells in the logarithmic growth phase were collected by trypsin digestion and configured into a 1 × 10^5^/mL cell suspension, which were inoculated in 96-well cell culture plates with 100 µL of cell suspension per well. They were incubated in a 37 °C, 5% CO_2_ incubator. When the cells were spread in a monolayer across the bottom of the well, the supernatant was removed, and 100 µL of the serum-free medium was added as the normal group, 100 µL of serum-free medium containing Lipopolysaccharide (LPS) (1 µg/mL) as the model group, and 100 µL of serum-free medium containing a final concentration of 50 µM GA, GA-SD and LPS (1 µg/mL) as the experimental group [28]. The culture was continued for 24 h, and the cell supernatant was collected to assess the levels of IL-1β, IL-4, IL-6, IL-10, MCP-1, TNF-α, IL-23 and IL-17A using enzyme-linked immunosorbent assay (ELISA) kits (Abcam) following the manufacturer’s instructions.

### 2.7. Construction of Mouse Ear Edema Model

The mice were randomly divided into 4 groups, each with 5 animals. These groups included the control group, TPA model group, GA group (20 mg/kg) and GA-SD group (20 mg/kg) (Table S2). All drugs were dissolved in DMSO, and the volume of DMSO was one-thousandth of the final solution volume and was diluted with PBS. All drugs were prepared before the assay. All mice, except the control and model mice, were administered at the same time each day, and the gavage operation lasted for one week. Normal and model mice were administered with the same volume of PBS. For ear edema, 1 h after the last gavage, TPA (0.3 µg) dissolved in 20 µL of acetone was repeatedly applied to the inner and outer surfaces of the mouse ears. The normal group was coated with an equal amount of acetone [29]. Orbital venous blood was taken from mice 6 h after the application of TPA for the detection of hepatorenal toxicity. Liver, kidney and ear tissues were taken for pathological section analysis.

### 2.8. Histopathology

To evaluate the therapeutic effect of GA and GA-SD on ear inflammation, ear tissues were collected from each group of mice, and to evaluate the hepatic and renal toxicity of GA and GA-SD, liver and kidney tissues were collected after perfusion and paraffin sectioning. The ear tissue and liver and kidney tissues were first fixed by immersion in 4% paraformaldehyde with gradient dehydration and paraffin embedding. They were sliced into 4 μm post-thin sections. They were stained with hematoxylin-eosin stain [30].

The sections were observed by optical microscopy, and three visual fields were randomly selected for analysis. Image analysis was performed by a pathologist who did not know the experimental conditions to evaluate the degree of tissue damage.

### 2.9. Analysis of Serum Biochemical Parameters

The orbital venous blood of mice in the normal group, the GA experimental group and the GA-SD experimental group were centrifuged at 3000 rpm for 10 min, and the serum was collected. According to the manufacturer’s instructions, the serum total protein (TP), serum albumin (ALB), creatinine (CREA), urea (UREA), aspartate aminotransferase (AST) and alanine aminotransferase (ALT) were measured with a commercial kit (CSCN). According to the normal reference values of the biochemical indices of Kunming mice provided by Huaying Biology, the biosafety of GA and GA-SD was evaluated.

### 2.10. Construction of Ethanol-Induced Gastric Ulcer Model

Animals were randomly assigned to five groups (*n* = 5) as follows: normal control group; model group; GA group (20 mg/kg) and GA-SD group (20 mg/kg) (Table S3). Mice in the normal control group and model group were administered with PBS. The GA and GA-SD samples were prepared according to the method described herein. Mice were administered by gavage once a day for 7 days. Before ulcer induction, animals fasted for 24 h and water was also withheld for two hours to empty their stomach of food. One hour after the last administration, mice except those in the normal control group were given 85% ethanol (10 mL/kg) by gavage to establish an acute gastric ulcer model [31].

### 2.11. Assessment of Gastric Tissue Injury and Histopathological Examination

One hour after the ulcer induction, animals were anesthetized with isoflurane and euthanized by cervical dislocation. The excised stomach was opened along the greater curvature and rinsed completely with PBS. Then, the stomachs were blotted dry with filter paper, spread out, and photographed. For the determination of gastric lesion area, the gastric ulcer index (GUI) was calculated by analyzing the inner surface of the stomach using Image J software (v1.53c.). The gastric tissues were taken for pathological section analysis after the photoshoot. For each group, the gastric ulcer index was determined using the following equation:

(1) $$\mathrm{Ulcer}\;\mathrm{Index}=\frac{\mathrm{Sum}\;\mathrm{of}\;\mathrm{lesion}\;\mathrm{areas}}{\mathrm{Total}\;\mathrm{stomach}\;\mathrm{area}}\times 100$$

The percentage of ulcer preventive index was calculated as follows:

(2) $$\mathrm{Preventive}\;\mathrm{index}=\frac{\mathrm{Ulcer}\;\mathrm{index}\left(\mathrm{ulcerated}\;\mathrm{group}\right)-\mathrm{Ulcer}\;\mathrm{index}\left(\mathrm{treated}\right)}{\mathrm{Ulcer}\;\mathrm{index}\left(\mathrm{ulcerated}\;\mathrm{group}\right)}\times 100$$

After a general examination, gastric tissues were preserved in a 4% paraformaldehyde buffer solution for 24 h and then embedded in paraffin wax. Slices were prepared and stained with hematoxylin and eosin (H&E) for histological evaluation.

### 2.12. Immunohistochemical (IHC) Staining

IHC staining was employed to assess the expression levels of pro-inflammatory cytokines TNF-α and IL-6. For a start, tissue sections were deparaffinized, rehydrated, and then antigen retrieved. Subsequently, the sections were pretreated for 25 min with sodium citrate buffer via heat-mediated antigen retrieval followed by treatment with 3% H_2_O_2_ to inhibit endogenous peroxidase activity and blocking with 3% BSA for 30 min. After that, the slices were treated with primary antibodies diluted at 1:500 overnight at 4 °C, and then with peroxidase-coupled secondary antibodies for 50 min. Color development was carried out by incubating with 3,3′-diaminobenzidine tetrahydrochloride for 5 min. The results were captured by light microscopy, followed by analysis by Image J software.

### 2.13. Statistical Analysis

All results are presented as the mean ± standard deviation (SD). Statistical comparison was made via Student’s *t*-test. The significance levels (*p* ˂ 0.05) were regarded as statistically acceptable.

## 3. Results

### 3.1. Preparation and Characterization of GA-SD

GA-SDs were prepared using the co-solvent evaporation method. As described in the experimental section, the GA and L-Arg solutions were mixed thoroughly and left for 30 min to form salts. Then, Soluplus^®^ was added, and Soluplus^®^ formed solid dispersions of the solutions via hydrogen bonding or complexation reactions.

**Figure 1:**
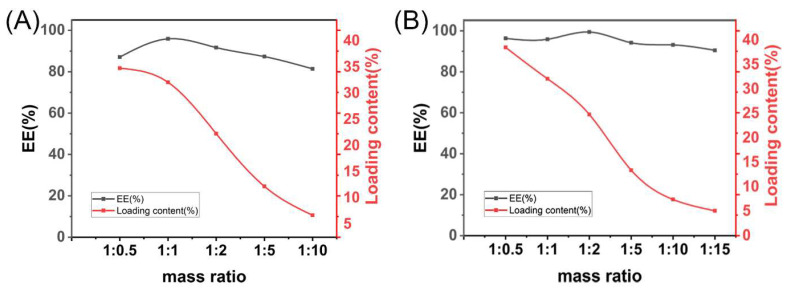
Formulation screening results. (**A**) The ratio of GA to Soluplus^®^ was fixed at 1:1 (*w*/*w*), and the ratio of L-Arg was changed. The mass ratio indicates the ratio of GA to L-Arg (*w*/*w*). (**B**) In the screening results shown in (**A**), the optimal ratio of GA to L-Arg was 1:1 (*w*/*w*), and based on this result, the ratio of Soluplus^®^ was changed; the mass ratio indicates the ratio of GA to Soluplus^®^ (*w*/*w*).

As shown in Figure 1A, the highest entrapment efficiency of the solid dispersion was achieved at 95.9% with a GA: L-Arg ratio of 1:1. A further increase in L-Arg resulted in decreased EE%, so the ratio of GA and L-ARG was most suitable at 1:1. The encapsulation efficiency was directly related to its bioavailability and in vivo efficacy. The highest value of entrapment efficiency (99.4%) was achieved at a mass ratio of 1:1:2 when the drug loading was 24.8% (Figure 1B). In aqueous solution, the polymer first formed hydrogen bonds with water to fully dissolve. When the GA: L-Arg: Soluplus^®^ was less than 1:1:2 (*w*/*w*/*w*), the encapsulation efficiency gradually increased to nearly 100%. At this point, we believed that GA-L-Arg was excessive and more Soluplus^®^ provided more loading space, leading to the increase in the encapsulation efficiency. As the Soluplus^®^ content increased, the entrapment efficiency did not increase further but instead decreased slightly, possibly because the excess dissolved Soluplus^®^ seized too many water molecules, resulting in there not being enough water to rehydrate with GA-Arg, making redissolving difficult [19].

The best mass ratio of GA, L-Arg and Soluplus^®^ was determined to be 1:1:2.

### 3.2. Physicochemical Characterization

#### 3.2.1. Particle Size and Zeta Potentials

**Figure 2:**
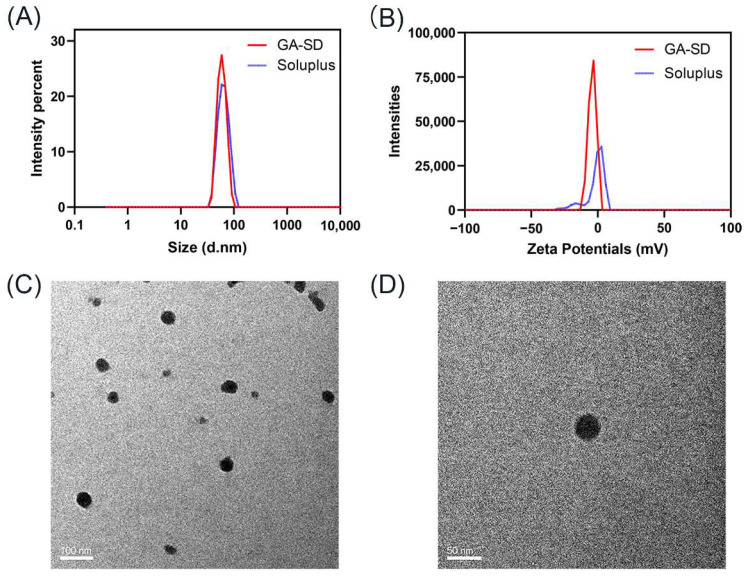
The particle size, Zeta Potentials and morphology of GA-SD (**A**) DLS analysis of GA-SD and Soluplus particle size. (**B**) Zeta Potentials analysis of GA-SD and Soluplus. (**C**,**D**) TEM analysis of GA-SD morphology.

In this experiment, the particle size and Zeta Potentials of Soluplus^®^ and prepared GA-SD were measured by DLS. The result is demonstrated in Figure 2A,B. It was known that Soluplus^®^ could form micelles spontaneously due to its amphipathic property. The results of zeta potential indicated that Soluplus^®^ micelles were formed with nearly neutral charges. Therefore, the charge of GA-SD should be derived from its loading components. We tested the potential of the GA-Arg complex and found that its charge was −3.4 mV, while GA-SD loaded a large number of complexes, which made the surface charge accumulate, showing the zeta potential was approximately −4.15 mV. An important factor in measuring the performance of nanoparticles is their particle size, which affects the circulation and biodistribution of the drugs [32]. The particle size of Soluplus^®^ was 63.17 nm, and the average particle size of GA-SD was 68 nm, which was very close to the particle size of Soluplus^®^. The DLS results of GA-SD showed a sharp peak, indicating single and narrow particle size distributions. Additionally, the microscopic details of GA-SD were confirmed by TEM (Figure 2C,D), and the GA-SD particles had irregular morphology, which was consistent with the basic characteristics of solid dispersions [22,32,33].

It has been reported that nanoparticles with a particle size below 100 nm can escape rapid clearance by the reticuloendothelial system, prolong the in vivo circulation time, increase cell membrane permeability, and improve bioavailability [34]. Therefore, the GA-SD prepared in this experiment may have higher cell membrane permeability and bioavailability than GA. In addition, liver cells are the main cells that phagocytose particles below 100 nm, so the hepatotoxicity of GA-SD had to be subsequently verified.

#### 3.2.2. XRD and FT-IR Analysis

**Figure 3:**
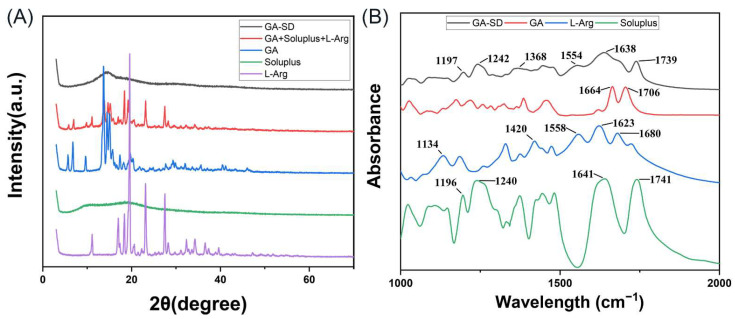
Structural analysis of solid dispersions. (**A**) XRD analysis of the GA-SD solid dispersion. (**B**) FT-IR spectra with the major characteristic peaks.

XRD and FTIR were used to characterize the interaction forms and the structure of the solid dispersion. The XRD results are plotted in Figure 3A. Figure 3A shows that GA and L-Arg were in crystalline form and Soluplus^®^ was an amorphous powder. Comparing the images, it can be seen that the characteristic peaks of both GA and L-Arg disappeared after the formation of solid dispersion, and a peak signal did not appear, suggesting an amorphous state of the powder which confirmed that GA was encapsulated in the Soluplus^®^. Thus, we successfully prepared GA-SD. Infrared spectroscopy can better analyze the molecular binding sites of GA, L-Arg and Soluplus^®^. The IR spectra of GA, L-Arg, Soluplus^®^, and GA-SD are plotted in Figure 3B and Figure S1. The absorption peak at 1664 cm^−1^ represented the stretching vibration of the C = O group of ketone segment in the GA structure, and the peak at 1706 cm^−1^ belonged to the stretching vibration of the C = O group of the carboxylic acid [16]. These signals disappeared completely in GA-SD, which might have been due to the encapsulation of the amphiphilic polymer Soluplus^®^. For Soluplus^®^, peaks around 1641 cm^−1^ and 1741cm^−1^ were related to the carbonyl groups in the vinyl acetate and caprolactam segments [35]. The infrared peaks of Arg were assigned according to the literature [36,37]. The peaks at 1680 and 1623 cm^−1^ were from the guanidyl group, including the C=N stretching, which reflected the basic functional group for salt formation with acidic compounds. The other peaks were assigned as follows: amide C–N stretching (1558 cm^−1^), COO– stretching (1420 cm^−1^), and NH_2_ deformation (1134 cm^−1^). Carboxylate anion usually shows an absorption peak at 1650–1550 cm^−1^ due to strong asymmetric carboxylate stretching [38]. The disappearance of the O-H stretches peak (3437 cm^−1^) of the carboxyl group in GA suggested the ionization of the carboxyl group [39,40,41]. There were two new peaks at 1554 cm^−1^ and 1368 cm^−1^, attributed to the asymmetric (Vas COO−) and symmetric stretching vibration (Vs COO−) of the –COO− group [16,42,43]. This indicated that interaction between GA and L-Arg led to the formation of a structure that belonged to a carboxylate.

The strong ionic interaction between the GA and L-Arg, the formation of salts and the hydrophilic structure of Soluplus^®^ make GA-SD extremely hydrophilic. Therefore, the water solubility of GA was greatly improved.

#### 3.2.3. Thermogravimetric Analysis

**Figure 4:**
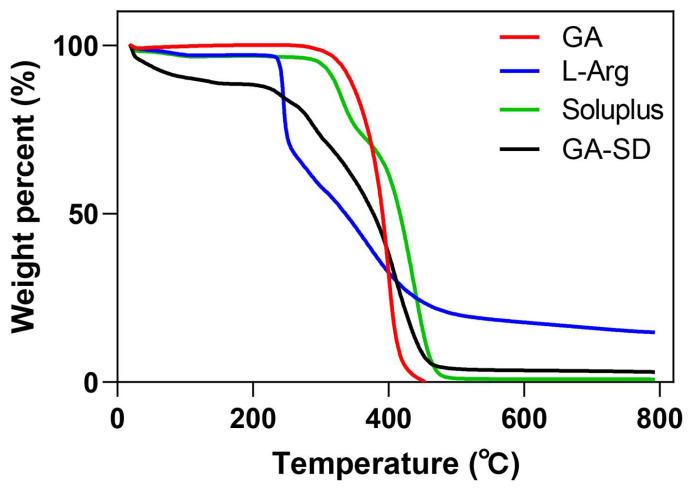
TGA curves of the mass loss as a function of temperature.

The TGA scan of GA showed almost a constant weight until 300 °C (Figure 4). GA began to decompose and lose weight as the temperature increased to 300 °C, which was close to the inherent melting point of GA at 294 °C. The weight loss of GA-SD in the early stage of heating may have been due to residual moisture from incomplete freeze-drying. Similar to Soluplus, GA-SD had no obvious endothermic peak, which indicated that the GA-SD was amorphous. The trend of GA-SD was very similar to Soluplus, which proved that Soluplus was contained in the outside of the GA. At 308 °C, the retention rate of GA-SD was 80%, which proved its good thermal stability.

#### 3.2.4. Stability of GA-SD

**Figure 5:**
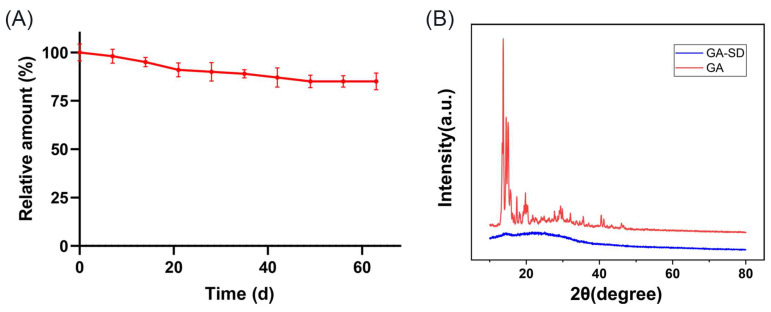
Stability of GA-SD. (**A**) Experimental results of GA-SD placement stability. Values are presented as the mean ± SD (*n* = 3). (**B**) Changes in the crystalline content of GA-SD after storage at 4 °C for 7 months.

The stability of GA-SD is shown in Figure 5A. GA-SD was placed at room temperature for 63 days, and the GA content only decreased by 9.8%, and the degradation rate was uniform. The results confirmed that GA-SD had good stability. Changes in the crystalline content of amorphous solid dispersions over time are shown in Figure 5B. Throughout all trials, the GA-SD preparation did not undergo crystallization, and even up to 7 months after preparation, dispersions still lacked any crystalline peaks by XRD, indicating that GA-SD had good crystalline stability. This may be related to the inherent properties of Soluplus^®^ and its crystallization inhibitory effect [44].

#### 3.2.5. Solubility GA-SD

The saturated solubility of GA in water was 6.62 × 10^−3^ mg/mL (Table 1), The detected saturation solubility was in good agreement with the reported 5.86 ± 0.45 × 10^−3^ mg/mL [15] and 6.36 × 10^−3^ mg/mL [45], and the saturated solubility of GA-SD in water was 37.6 ± 0.03 mg/mL. The dissolution phenomenon was shown in Figure S2. Compared with the GA group, the GA-SD group possessed a higher dissolving ability with a 5680-fold higher dissolving ability in water. The solubility of the drug is critical to the efficacy of the drug. The excellent solubility of GA-SD may improve the absorption and bioavailability of GA in oral administration.

### 3.3. Anti-Inflammatory Effect In Vitro

**Figure 6:**
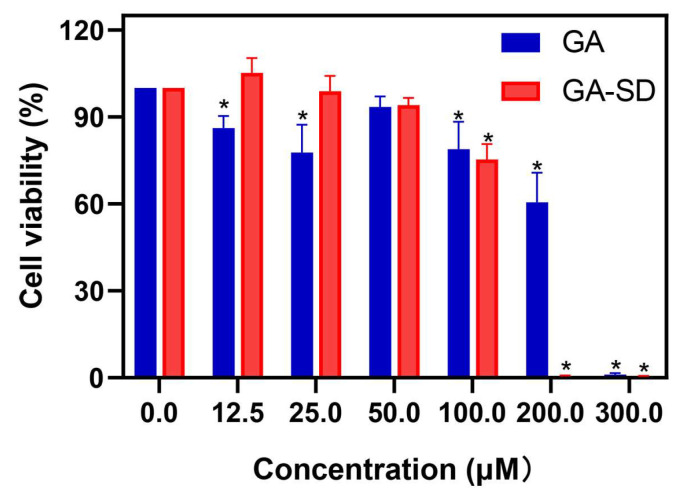
Cell viability at different drug concentrations on RAW 264.7 cells. The values presented are the means ± SD (*n* = 4). 0 μM represents the control group. * *p* < 0.05 vs. Control.

To further study the bioavailability of GA-SD in vivo, we examined the effect of this material on the anti-inflammatory activity of GA. In this study, we finally determined the safe administration concentration to be 50 µM, at which RAW 264.7 cells maintained high viability (>90%). The result of the cell viability assay is shown in Figure 6.

**Figure 7:**
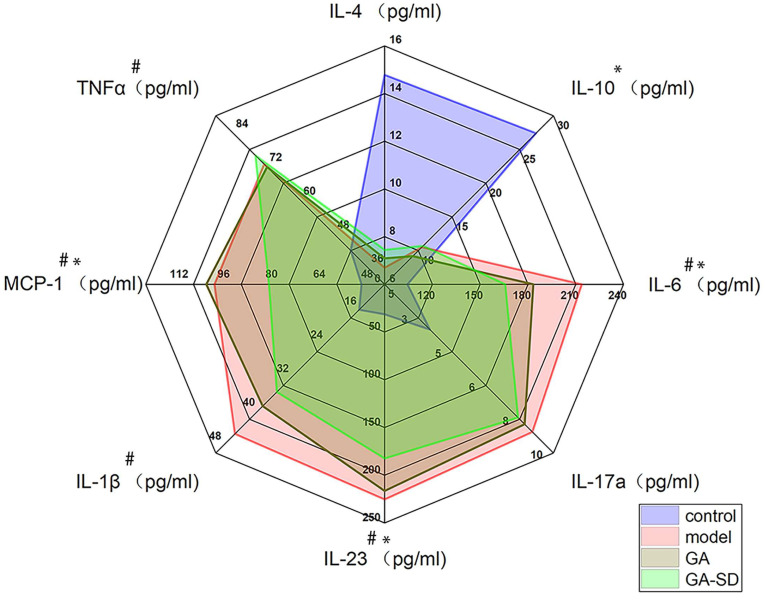
Effect of GA and GA-SD on LPS-induced secretion levels of cytokines such as IL-4, IL-1β, IL-6, MCP-1, TNF-α, IL-23, IL-17A, and IL-10 in RAW264.7 cells. Values are presented as the mean ± SD (*n* = 3). # *p* < 0.05 vs. model group. * *p* < 0.05 vs. LPS group.

LPS stimulation of the mouse peritoneal mononuclear macrophage cell Line 264.7 was used to simulate the inflammatory response in vitro, and the cell supernatant was analyzed for inflammatory factors by ELISA. When cells are stimulated by LPS, the secretion of proinflammatory cytokines, such as IL-1β, IL-6, MCP-1, TNF-α, IL-23, and IL-17A, increases, while the secretion of anti-inflammatory cytokines such as IL-4 and IL-10 decreases. Cytokines, such as IL-1β, TNF-α, and IL-6, are key proinflammatory factors that can mediate inflammation by inducing cellular pro-inflammatory gene expression [46,47]. IL-10 and IL-4 are key anti-inflammatory factors that inhibit the production of pro-inflammatory molecules, limit excessive immune responses, and play a key role in the treatment of inflammation [48,49]. The experimental results are shown in Figure 7. After treatment with free GA and GA-SD, the release of pro-inflammatory cytokines, due to LPS stimulation, was inhibited by free GA and GA-SD. The secretion of anti-inflammatory cytokines was elevated. Moreover, the immunomodulatory effect of GA-SD treatment was superior to that of the free GA experimental group. GA-SD protects the anti-inflammatory activity of free GA itself, and the enhanced anti-inflammatory effects might be due to the presence of a solubility enhancer (Soluplus^®^), leading to higher solubility. Moreover, the suitable particle size of GA-SD enhanced the permeability of its cell membrane, which also improved the utilization of the drug by the cells and enhanced the anti-inflammatory effect.

### 3.4. Anti-Inflammatory Efficacy on TPA-Induced Mouse Ear Edema

**Figure 8:**
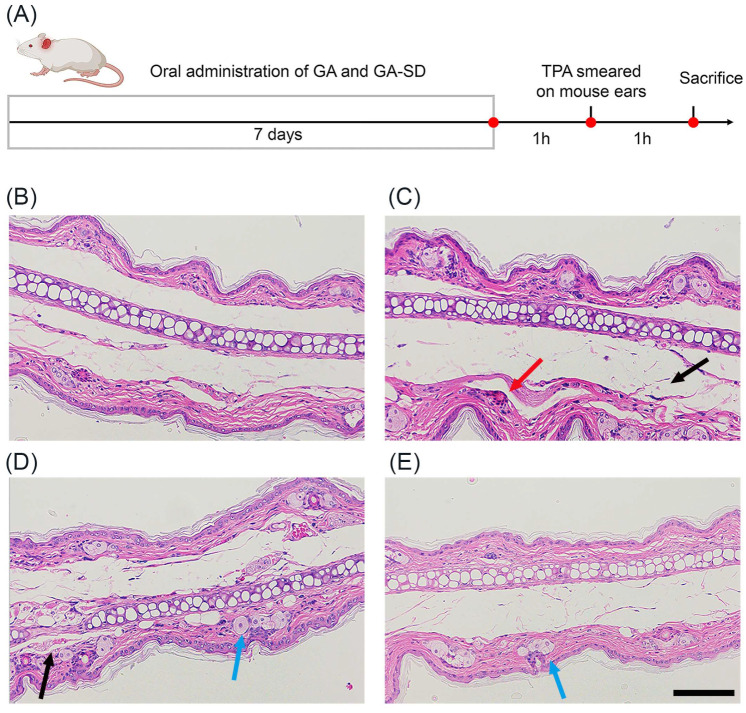
Histopathology of mouse ear in each experimental group. (**A**) Experimental scheme of TPA-induced mouse ear edema model. H&E staining of ear tissue slices after treatment with (**B**) PBS+ acetone, (**C**) PBS+TPA, (**D**) free GA+TPA, and (**E**) GA-SD+TPA. Scale bar: 100 μm. The black arrow marks the discrete rupture of the transverse muscle, the red arrow marks the small vessel congestion, and the blue arrow marks the inflammatory cell infiltration.

Based on the current anti-inflammatory effect of solid dispersion GA-SD in vitro, this study was conducted to construct a mouse ear edema model by applying TPA to the outer part of the mouse ear, and the anti-inflammatory effect of GA-SD in vivo was evaluated by gavage administration, using free GA in the control group. The experimental scheme (Figure 8A) and the hematoxylin-eosin staining (H&E) results of the ears of mice receiving different drug treatments are shown in Figure 8. Compared with the ear tissues of the control group of mice (Figure 8B), those of the TPA model group (Figure 8C) showed increased thickness of the auricle, discrete breaks in the transverse muscle fibers, partial congestion of small blood vessels was visible, and a significant increase in inflammatory cell infiltration. In the GA-SD treatment group (Figure 8E), ear tissue edema and transverse muscle fiber breaks were significantly reduced, as was the number of infiltrating inflammatory cells. The inflammatory response induced by TPA was effectively suppressed. In the free GA-treated group (Figure 8D), transverse muscle rupture was reduced, but inflammatory cell infiltration and small vessel congestion were not effectively suppressed. The GA-SD enhanced the anti-inflammatory effect of free GA in vivo. The enhanced anti-inflammatory effect was closely related to the increased solubility of free GA by GA-SD. As a nano-drug delivery system of less than 100 nm, solid dispersions can be adsorbed by the adsorption endocytosis pathway [50,51]. The enhanced cell membrane permeability increased the absorption of GA by the gastrointestinal tract and further increased oral bioavailability for better in vivo anti-inflammatory effects.

### 3.5. The Biosafety of GA-SD

#### 3.5.1. Histopathological Results from the Liver and Kidney

**Figure 9:**
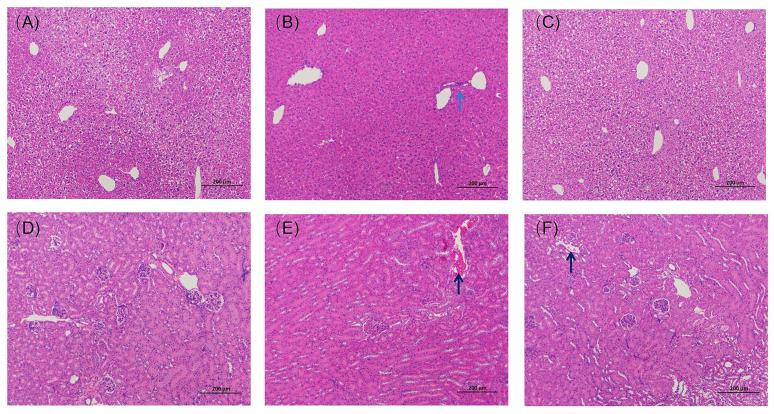
Histopathological studies of the liver and kidney of each group of mice after gavage administration. (**A**) Liver tissue section of the control group; (**B**) liver tissue section of the free GA administration group; (**C**) liver tissue section of the GA-SD administration group; (**D**) kidney tissue section of the normal group; (**E**) kidney tissue section of the GA administration group; (**F**) kidney tissue section of the GA-SD administration group. Scale bar: 200 μm. Black arrows mark cellular debris in the renal tubules; blue arrows mark the phenomenon of inflammatory cell infiltration.

Arg is a unique, non-toxic, and biocompatible biomolecule [52]. Parikh A, et al. [53] confirmed that histopathological examinations of all the vital organs did not reveal any treatment-related changes in a Soluplus-treated mouse group, indicating the material Soluplus^®^ is biocompatible and could be a suitable candidate for orally administered dosage forms [54]. GA-SD possesses unique physicochemical properties and further improves in vivo cellular uptake efficiency by modifying the free GA morphology and size, but it may also affect the biosafety of the solid dispersion. Thus, a detailed evaluation of GA-SD’s potential toxicity in vivo must precede any prospective applications. The results of H&E staining of the liver tissues of mice treated with different drugs are shown in Figure 9A–C. The results showed that the liver tissues of the GA-SD-treated mice were similar to those of the normal group mice. There were abundant numbers of hepatocytes with neatly arranged hepatic cords and intact structures, and no focal necrosis of hepatocytes, fat droplets, or obvious inflammatory cell infiltration was observed. A small amount of inflammatory cell infiltration was seen in the liver tissue of mice in the free GA-treated group, though there was no other obvious pathological damage. The H&E staining results of the kidney tissues of mice receiving different drug treatments are shown in Figure 9D–F. They showed that the kidney tissues of GA-SD-treated mice were similar to those of the control group, with normal glomerular size, intact basement membranes, no obvious inflammatory cell infiltration, and a small number of cellular debris visible in the renal tubules. A certain amount of cellular debris was present in the renal tubules of the mice in the free GA-treated group, though no other obvious damage was observed. This result confirmed that there was no significant histopathological difference between the GA-SD-treated and normal groups and that GA-SD had good biosafety. 

#### 3.5.2. Effect of GA-SD on Liver and Kidney Function

**Figure 10:**
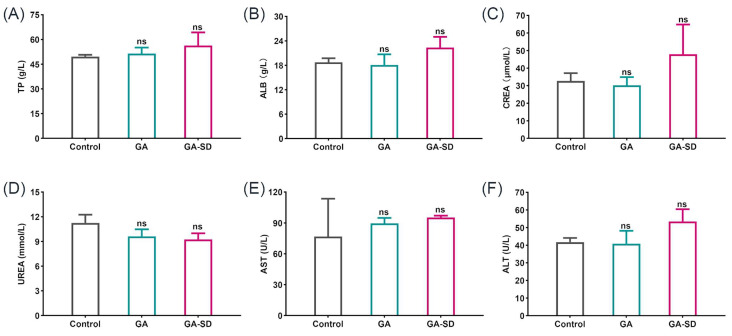
(**A**–**F**) Serum biochemical changes of drugs treated mice. Values are presented as the mean ± SD (*n* = 3). ns *p* > 0.05 vs. control group. Biochemical index reference values: TP (57.1 ± 19.4 g/L), ALB (22.4 ± 5.1 g/L), CREA (50.4 ± 35.4 μmol/L), UREA (8.99 ± 2.89 mmol/L), AST (<400 U/L), and ALT (<400 U/L).

The liver is the main target organ for toxic compounds, while the kidney is a metabolic organ with multiple functions, such as eliminating blood waste and balancing body fluids. In this study, we examined alterations in liver and kidney function biomarkers (AST, ALT, TP, ALB, CREA, UREA) in male Kunming mice after oral administration of GA and GA-SD, thus, assessing the effects of GA and GA-SD on liver and kidney function. Increase in liver enzymes is related to the change in cell membrane permeability caused by liver and hepatocyte damage, so the change in liver enzyme content reflects the hepatotoxicity of the drug. Creatinine and urea are important indicators used to reflect the detoxification of the kidneys, by which the status of renal function is judged as normal [55,56]. The biochemical parameters (TP, ALB, CREA, UREA, AST and ALT) are shown in Figure 10A–F. According to the results, the serum levels of ALT, ACT, TP, ALB, CREA, and UREA in the GA-SD mice were within the normal index range [57,58], and there were no significant changes in the levels of each biochemical index compared to the normal group. The serum biochemical levels were consistent with the histopathological results of the kidney and liver. This result suggested that GA-SD administration did not cause abnormal liver and kidney functions. Therefore, GA-SD has good biosafety.

### 3.6. Anti-Inflammatory Efficacy on Ethanol-Induced Gastric Ulcer Model

#### 3.6.1. Effect of GA-SD on the Pathological Features of Ethanol-Induced Gastric Ulcer Model

**Figure 11:**
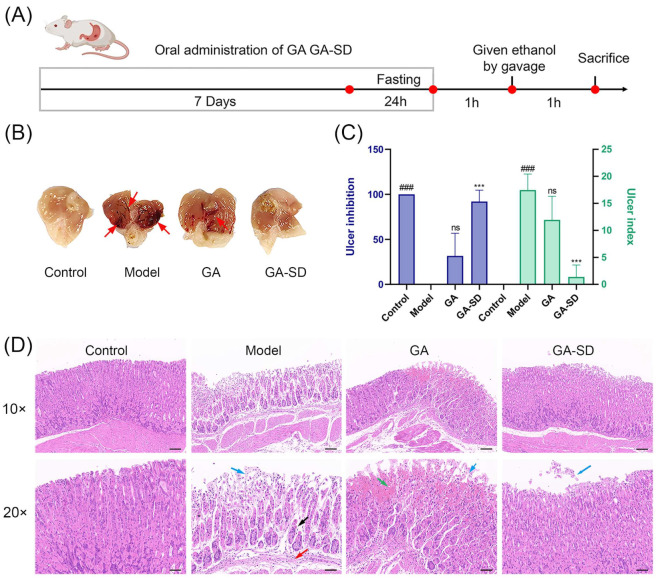
Effect of GA-SD on ethanol-induced gastric ulcer in mice. (**A**) Experimental protocol. (**B**) Macroscopic appearance of gastric tissues (**C**) Ulcer index and the inhibition rate. Data are presented as the mean ± SD (*n* = 3). Different letters indicate statistically significant differences at the level of *p* < 0.05. ^###^ *p* < 0.001 when compared with the control group; *** *p*  <  0.001 when compared with the model group. (**D**) H&E staining of gastric tissues (magnification of 10× and 20×). Scale bar of 10×: 100 μm. Scale bar of 20×: 50 μm. Green arrow: hemorrhagic injury; blue arrow: loss of gastric epithelial cells; red arrow: inflammatory cells infiltration; black arrow: disorganized glandular structure.

To further evaluate the anti-inflammatory effect of GA-SD, we established an ethanol-induced gastric ulcer model (Figure 11A). One hour after EtOH gastric ulcer induction, the gastric mucosa of mice in the normal control group was smooth and light pink, also lacking submucosal edema and hemorrhage (Figure 11B and Figure S3). In contrast, the model group gastric mucosa showed severe damage with linear and striated hemorrhagic ulceration covered with coagulated blood, submucosal edema, and erosion. Compared with the model group, bleeding of gastric mucosa was improved in the GA group, but linear hemorrhagic ulceration was still present (ulcer index: 11.94; ulcer inhibition rate %: 31.67) (Figure 11C). The gastric mucosa of GA-SD treated mice were the same as those in the normal control group, with light pink and almost no ulceration (ulcer index:1.38; ulcer inhibition rate %:92.07). The local and systemic inflammatory response is common in patients with gastric ulcers. The degree of inflammatory response is directly related to the severity of gastric ulcers. The result shows that GA-SD could significantly improve the damage to gastric tissue and reduce the number of bleeding points, suggesting that GA-SD may have a protective effect against gastric ulcer induced by ethanol in mice. Some novel formulations are expected as anti-inflammatory agents to increase the oral uptake of GA. The GA-SD enhanced the anti-inflammatory effect of free GA in vivo.

#### 3.6.2. Histopathological Examination of Gastric Tissues

The results of hematoxylin and eosin-stained (H&E) gastric sections further verified the enhanced anti-inflammatory effect of GA-SD (Figure 11D). Examination of the control group showed normal architecture with intact mucosa, submucosa, muscularis and serosa. In contrast, ethanol caused extensive damage to the gastric tissue. We observed focal areas of erosions and ulcers with epithelial loss, irregular arrangement of gastric glands, the decrease of mucosal glands, and the infiltration of submucosal inflammatory cells. In the GA group, we observed mild submucosal edema, epithelial loss, and a small area of gastric gland necrosis with hemorrhage in the gastric tissue. Stomachs of the GA-SD group showed nearly normal gastric architecture with the absence of epithelial erosion, submucosal edema, and leucocyte infiltration. Ulcered mice, treated with GA-SD demonstrated comparatively better protection of the stomach compared to those treated with GA. The better anti-ulcer and anti-inflammatory effects of GA-SD compared to GA were confirmed by histological analysis. The enhanced anti-inflammatory effect is closely related to the increased solubility of free GA by GA-SD. 

#### 3.6.3. Immunohistochemical Analysis of Gastric Tissues

**Figure 12:**
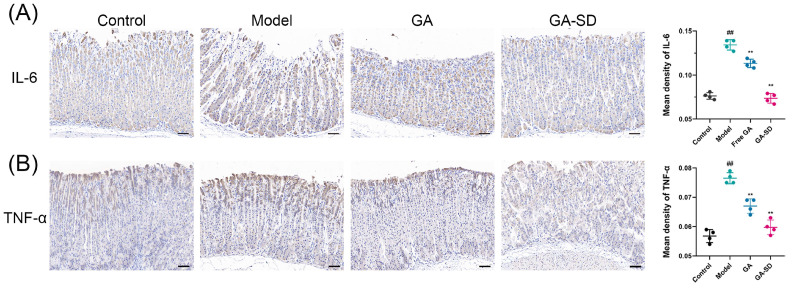
Immunohistochemical analysis of IL-6 and TNF-α in gastric tissues (magnification of 20×). Scale bar: 50 μm. (**A**) Representative photomicrographs of immunohistochemical staining for IL-6 among different groups. (**B**) Representative photomicrographs of immunohistochemical staining for TNF-α among different groups. Data are presented as the mean ± SD (*n* = 4) ^##^ *p* < 0.01 vs. control. ** *p*  <  0.01 vs. the model group.

Gastric mucosa damage activates the inflammatory process, which increases the secretion of inflammatory cytokines, leading to gastric mucosal damage [1,59,60]. Pro-inflammatory cytokines in the tissue may be used as biomarkers of gastric visceral damage. The overexpression of TNF-α and IL-6 is considered deleterious to the gastrointestinal system, as it promotes the accumulation of neutrophils, lymphocytes and monocytes/macrophages at inflammatory sites, thereby disrupting the mucosal barrier [61,62,63,64]. The protein expression of TNF-α and IL-6 were stained in Cytoplasm, and the positive expression of TNF-α and IL-6 in the ethanol ulcerated group was significantly higher than that in the other two groups (Figure 12). The increased levels of TNF-α and IL-6 in the gastric tissues of ethanol ulcerated mice were confirmed via IHC staining. Compared with the ethanol ulcerated group, the GA-SD group significantly reduced the release of TNF-α and IL-6. Collectively, GA-SD was more effective than free GA in preventing ethanol-induced acute gastric ulcer, which was related to the improved solubility and bioavailability of GA-SD and enhanced anti-inflammatory activity.

## 4. Conclusions

In this study, we used the co-solvent evaporation method to make GA and L-Arg react to form salts and then added the polymer-solvent Soluplus^®^ with an amphiphilic chemical structure. GA-SD solid dispersions with particle size less than 100 nm, irregular morphology and good stability were successfully prepared. GA-SD has a particle size below 100 nm, which allows GA-SD to avoid rapid clearance by the reticuloendothelial system and increases cytomembrane penetrability. The presence of L-Arg and Soluplus^®^ in the solid dispersion enables GA-SD to have high solubility; therefore, many of the limitations of free GA, such as poor water solubility, low cellular utilization, and low bioavailability, have been solved. The results of anti-inflammatory effect in vitro showed that the immunomodulatory effect of GA-SD was superior to free GA and GA-SD enhanced the anti-inflammatory effect of free GA in vitro. The anti-inflammatory effect of GA-SD in vivo was evaluated by gavage administration in a TPA-induced mouse ear edema model and ethanol-induced gastric ulcer model. The results indicated that GA-SD effectively improved the anti-inflammatory effect of free GA in vivo. Furthermore, GA-SD had no significant effects on liver and kidney functions, there was no significant tissue toxicity and it displayed good biosafety. With the improvement of the solubility and utilization of GA, the application scope of GA in the treatment of many inflammatory diseases will be expanded. As a safe and effective solid dispersion, it is a promising anti-inflammatory oral formulation and may provide some references for other oral drug candidates with low bioavailability.

## Figures and Tables

**Table 1:** Saturated solubility of GA and GA-SD.

Group	GA	GA-SD
Saturated solubility(mg/mL)	6.62 × 10^−3^	37.6 ± 0.03

Values are presented as the mean ± SD (*n* = 3).

## Data Availability

The data presented in this study are available in the article or Supplementary Materials here.

## Supplementary Materials

The following supporting information can be downloaded at: https://www.mdpi.com/article/10.3390/pharmaceutics14091797/s1, Table S1: Relative proportions of solid dispersion components; Table S2: Grouping and sampling of mice in the ear edema model (*n* = 5). Table S3: Grouping and sampling of mice in ethanol-induced gastric ulcer model (*n* = 5); Figure S1. FT-IR analysis; Figure S2. Dissolution phenomena for 10 mg GA, GA-SD samples in 1 mL water; Figure S3. Macroscopic appearance of gastric tissues.
