# Supplementary material for: Enhancement of the Bioavailability and Anti-Inflammatory Activity of Glycyrrhetinic Acid via Novel Soluplus®—A Glycyrrhetinic Acid Solid Dispersion
Source: Pharmaceutics. 2022 Aug 26;14(9):1797. doi: 10.3390/pharmaceutics14091797 (PMC9504515; doi:10.3390/pharmaceutics14091797)
Supplement: Supplementary file 1 [file pharmaceutics-14-01797-s001.zip › pharmaceutics-1790737-SI.pdf]

Table S1. Relative proportions of solid dispersion components.

GA	L-Arg	Soluplus®
1	0.5	1
1	1	1
1	2	1
1	5	1
1	10	1
1	1	0.5
1	1	2
1	1	5
1	1	10
1	1	15

Note: Reagent ratios are presented by the mass ratio (w/w/w)

Table S2. Grouping and sampling of mice in the ear edema model (n=5).

Group	TPA-induced	Gavage drug delivery	Tissue Sampling
Control	-	PBS	Ear, Liver, Kidney
TPA group	+	PBS	Ear
GA group	+	GA (20 mg/kg)	Ear, Liver, Kidney
GA-SD group	+	GA-SD (20 mg/kg)	Ear, Liver, Kidney

Table S3. Grouping and sampling of mice in ethanol-induced gastric ulcer model (n=5).

Group	ethanol-induced	Gavage drug delivery	Tissue Sampling
Control	-	PBS	Stomach
Model group	+	PBS	Stomach
GA group	+	GA (20 mg/kg)	Stomach
GA-SD group	+	GA-SD (20 mg/kg)	Stomach

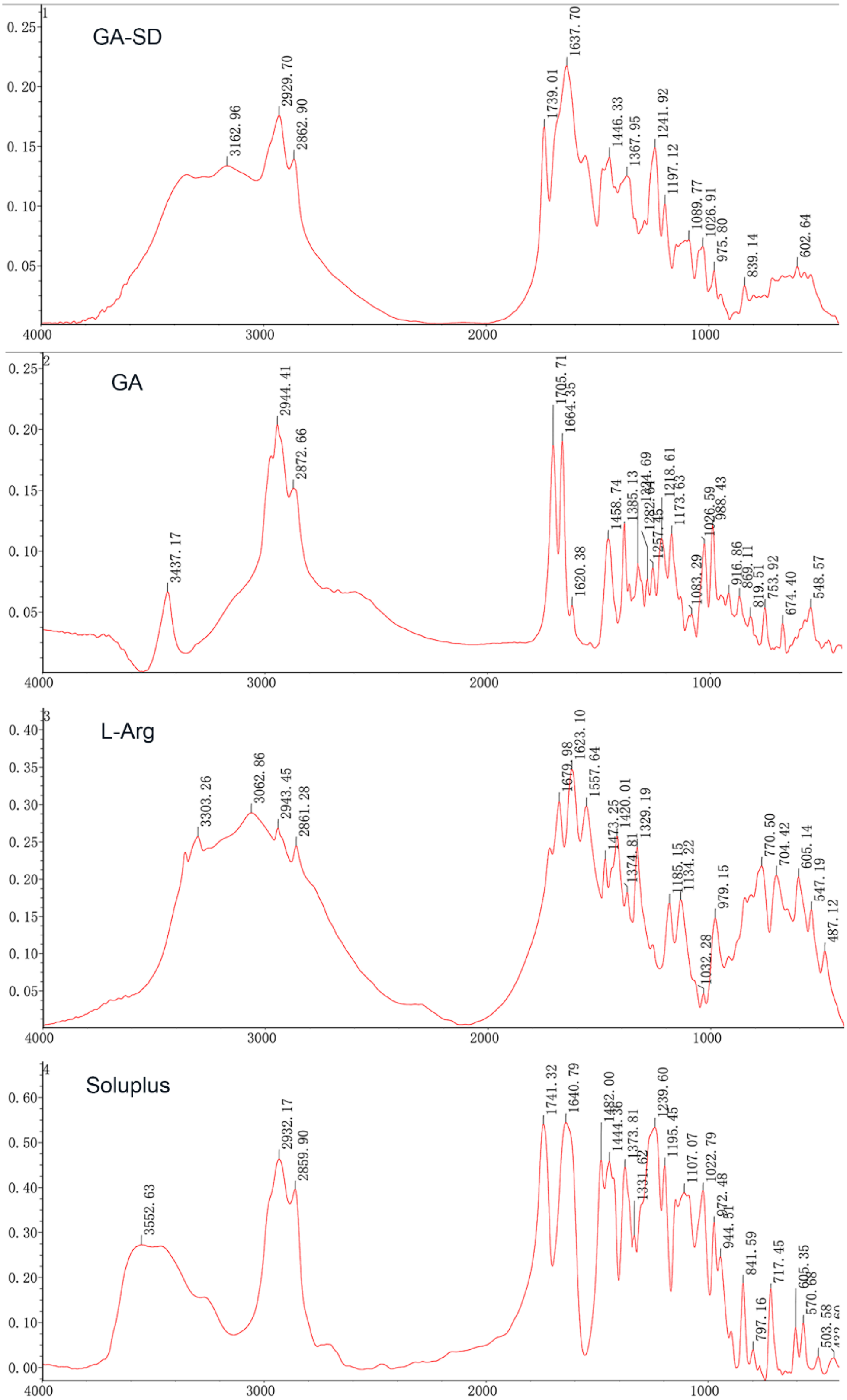

Figure S1. FT-IR analysis.

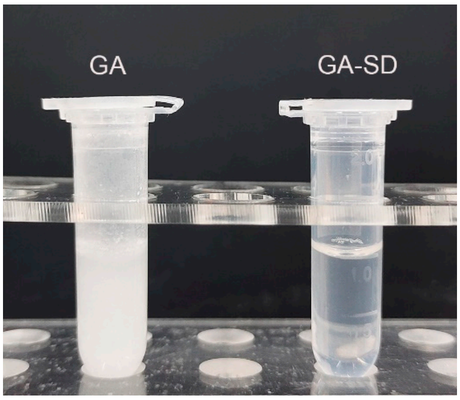

Figure S2. Dissolution phenomena for 10 mg GA, GA-SD samples in 1 mL water.

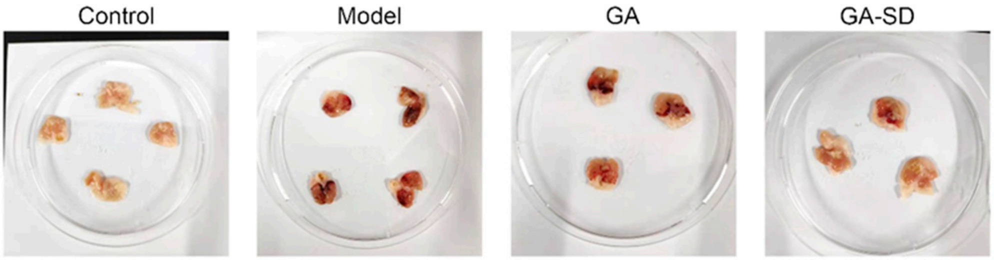

Figure S3. Macroscopic appearance of gastric tissues.
